# Discrete Hedgehog Factor Expression and Action in the Developing Phallus

**DOI:** 10.3390/ijms21041237

**Published:** 2020-02-12

**Authors:** Gerard A. Tarulli, Andrew J. Pask, Marilyn B. Renfree

**Affiliations:** School of BioSciences, The University of Melbourne, Parkville, VIC 3010, Australia; a.pask@unimelb.edu.au (A.J.P.); m.renfree@unimelb.edu.au (M.B.R.)

**Keywords:** hypospadias, urethra, penis, bone, hedgehog signalling

## Abstract

Hypospadias is a failure of urethral closure within the penis occurring in 1 in 125 boys at birth and is increasing in frequency. While paracrine hedgehog signalling is implicated in the process of urethral closure, how these factors act on a tissue level to execute closure itself is unknown. This study aimed to understand the role of different hedgehog signalling members in urethral closure. The tammar wallaby (*Macropus eugenii*) provides a unique system to understand urethral closure as it allows direct treatment of developing offspring because mothers give birth to young before urethral closure begins. Wallaby pouch young were treated with vehicle or oestradiol (known to induce hypospadias in males) and samples subjected to RNAseq for differential expression and gene ontology analyses. Localisation of Sonic Hedgehog (SHH) and Indian Hedgehog (IHH), as well as the transcription factor SOX9, were assessed in normal phallus tissue using immunofluorescence. Normal tissue culture explants were treated with SHH or IHH and analysed for *AR*, *ESR1*, PTCH1, GLI2, *SOX9*, *IHH* and *SHH* expression by qPCR. Gene ontology analysis showed enrichment for bone differentiation terms in male samples compared with either female samples or males treated with oestradiol. Expression of SHH and IHH localised to specific tissue areas during development, akin to their compartmentalised expression in developing bone. Treatment of phallus explants with SHH or IHH induced factor-specific expression of genes associated with bone differentiation. This reveals a potential developmental interaction involved in urethral closure that mimics bone differentiation and incorporates discrete hedgehog activity within the developing phallus and phallic urethra.

## 1. Introduction

Disorders in the development of reproductive organs are the most common birth defects worldwide. Among them, hypospadias effects 1 in 250 births [1,2] and is a defect in urethral closure so that the opening is positioned within the posterior penile meatus rather than the tip of the glans penis. Alarming increases in incidence of up to 2% a year have been reported [1,2], that highlights the current burden of hypospadias and the value in understanding its underlying causes.

While it is known that gene regulatory networks critical for bone/limb development play important roles in regulating phallus development and urethral closure [3,4,5,6], it is yet to be determined how these factors are coordinated on a tissue level to execute the process of urethral closure itself. One factor important in bone and limb development is the secreted signalling factor sonic hedgehog (SHH). SHH plays important roles in the development of the genital tubercle from which the mouse phallus forms [7,8,9,10] as well as later masculinisation events and the formation of the penile urethra [8,11]. Published evidence indicates that expression of SHH in urethral epithelial cells signals via underlying mesenchyme to promote proliferation and growth of the genital tubercle and urorectal septum mesenchyme [8]. The importance of hedgehog signalling is conserved in humans, as evidenced by polymorphisms in hedgehog pathway genes being associated with an increased risk of delivering a baby with hypospadias [12]. A second hedgehog family member implicated in urethral closure and phallus development is Indian hedgehog (IHH). Far less is known about the role of IHH in this process, though its expression is positively regulated by androgens, and knockout of IHH results in a failure of proper phallus masculinisation in the mouse [13]. However, whether these hedgehog factors have distinct functions in urethral closure has never been investigated.

The importance of androgens in phallus development and urethral closure has a long evolutionary history [3] and is well described in literature [14,15,16,17,18,19]. The process of urethral closure is highly sensitive to androgen disruption during a critical window of developmental programming that precedes the initiation of closure [20,21,22]. Therefore factors that can interfere with normal androgen signalling during this critical window, such as oestrogenic chemicals, are associated with hypospadias [14,23,24]. Making direct assessments of the gene regulatory changes that occur in response to hypospadias-inducing chemicals during this programming window are key to capturing the fundamental genetic networks underpinning urethral closure that go awry to contribute to hypospadias. In most species this programming window occurs during fetal development and therefore most animal models of chemical-induced hypospadias use indirect assessments via treating the mothers during pregnancy. This introduces confounding factors such as maternal metabolism and placental transfer that complicate experimental analysis.

One way to overcome these limitations and perform direct assessments in vivo is to use a marsupial model such as the tammar wallaby (*Macropus eugenii*). Marsupials give birth to altricial young, and in the tammar wallaby this occurs before androgen programming and urethral closure begin. This allows for offspring to be treated directly while in the pouch, for the entire duration of androgen programming. The tammar wallaby model was used to identify the gene regulatory networks most enriched when wallaby offspring are treated with ethinylestradiol (E2), a known hypospadias-inducing chemical in this species, during the critical androgen programming window. Comparisons between male and female phallus tissue were also made. Findings of this study identified regulation of ossification and osteoblast differentiation as being the most highly enriched gene ontologies altered by E2, and when comparing normal male and female phallus tissue. Further analysis showed, for the first time, the tissue region-specific expression of different hedgehog factors that we propose is akin to the discrete spatial functions of specific hedgehog factors in developing bone.

## 2. Results

### 2.1. Enrichment of Bone Differentiation Ontologies between Male and Female Phallus Tissue, or Males Treated with Oestradiol

**Figure 1:**
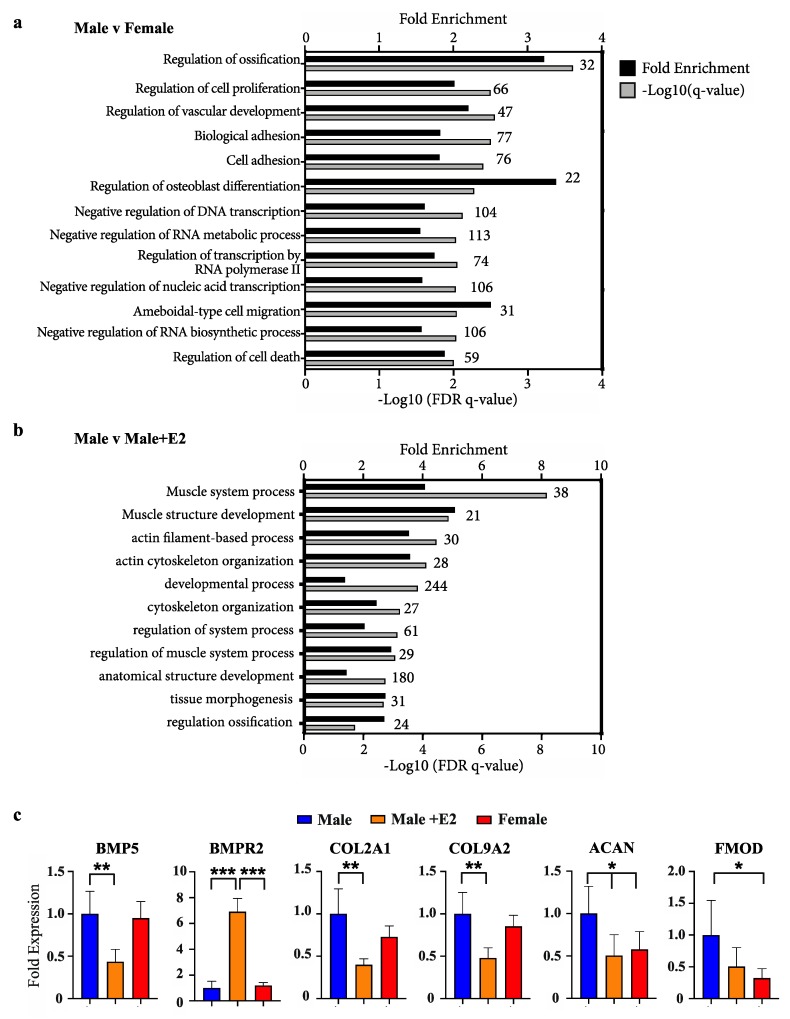
Enrichment of bone development ontologies in male versus female and males treated with oestradiol (**a**) Fold enrichment (black bars) and FDR q-values (grey bars) of process gene ontologies from differentially expressed genes between male and female phallus, or (**b**) male phallus with and without oestradiol treatment. Numbers above bars on graph indicate the number of enriched genes in that ontology (**c**) Representative bone/chondrocyte development genes and their relative levels in RNAseq data. (*n* = 5 animals/group; mean +/− s.d, * = <0.05, ** = <0.01, *** = <0.001).

Ranked lists of differentially expressed genes from outputs of DESeq2 analysis were assessed by gene ontology using GOrilla [25]. Due to the non-conventional animal model employed and the incomplete annotation of the wallaby genome available in these studies, only 40% of gene IDs were recognised in GOrilla analysis. Comparing male and female phallus tissue, all ontologies with significant enrichment by a false-discovery *q*-value <0.01 are listed in Figure 1a. The most highly enriched ontology was regulation of osteoblast differentiation (3.4-fold) and regulation of ossification (3.2-fold). Additional ontologies of significance include those relating to gene transcription, as well as cell proliferation, migration and adhesion. Gene ontology analysis for differentially expressed genes between male phallus tissue and male phallus tissue from pouch young treated with E2 are listed in Figure 1b. Muscle system process was the most significantly enriched ontology in this comparison, followed by other ontologies associated with muscle and cytoskeletal processes. Regulation of ossification was also enriched, though with a lower significance compared with enrichments in Figure 1a.

Examples of known bone differentiation-associated genes and expression changes from RNAseq data are shown in Figure 1c. Significant changes in a fundamental bone development factor and receptor, bone-morphogenic protein 5 (BMP5) and BMP receptor (BMPR2) were observed when comparing males with males treated with E2. Additionally, significant changes in chondrocyte differentiation markers (COL2A1 and COL9A2) and functional mediators of bone development (aggrecan (ACAN) and fibromodulin (FMOD) were also observed.

### 2.2. Expression of Specific Hedgehog Factors Was Spatially Restricted in the Developing Phallus

**Figure 2:**
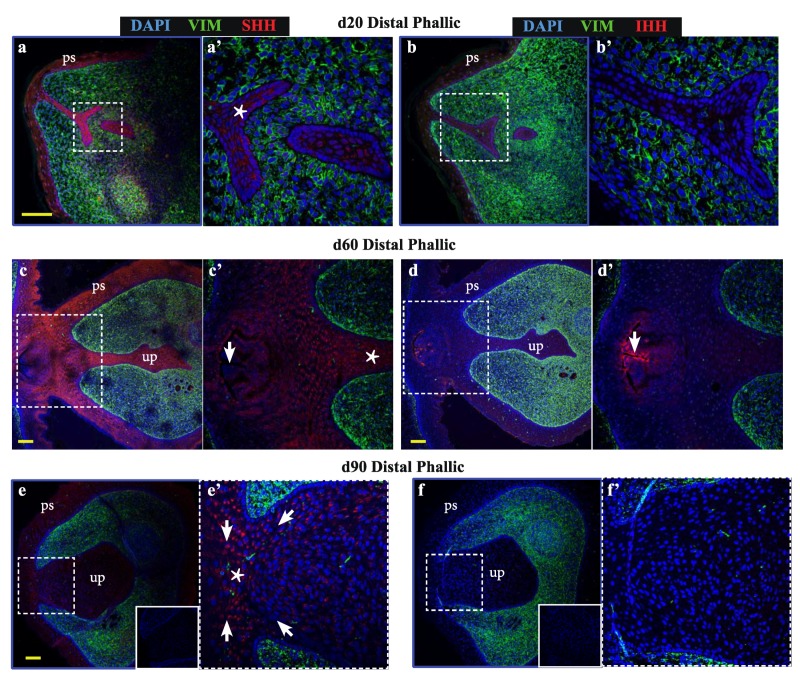

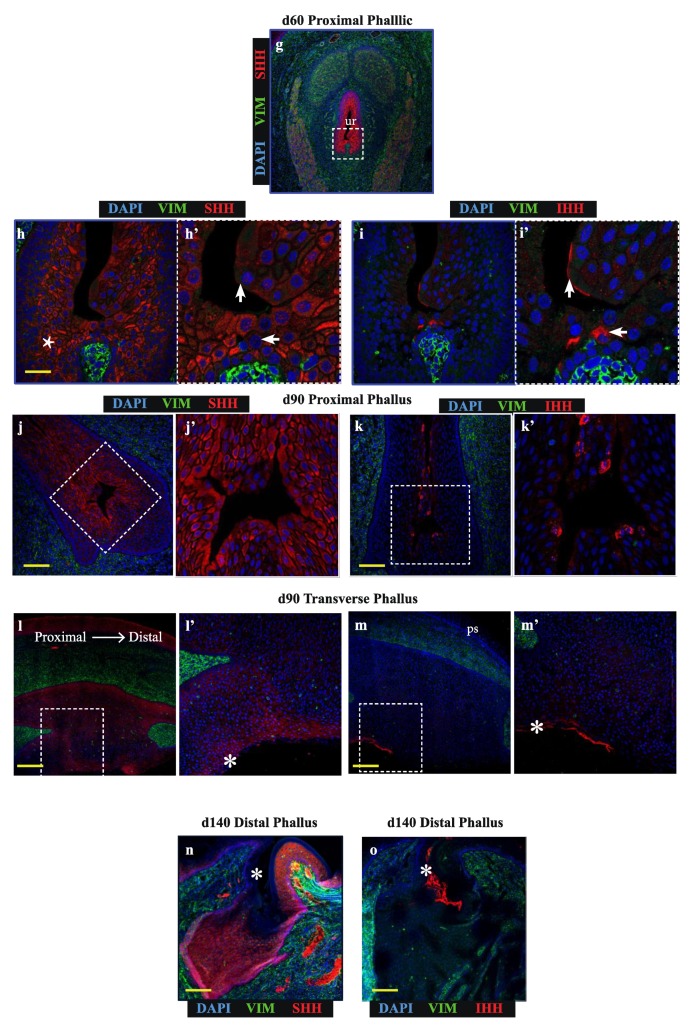
Sonic hedgehog (SHH) and Indian hedgehog (IHH) localize to discrete regions of the developing phallic epithelium. Day 20 male phallus immunolabelled for SHH (red—a, c, e, g, h, j) or IHH (red—b, d, f, i, k) and vimentin (green—all images). Dotted box denotes magnified portion in adjacent image. The expression of SHH in the distal phallic epithelium at day 20 (**a**), day 60 (**c**) and day 90 (**e**) is extensive in urethral plate epithelium (up) and phallic skin (ps). Cell direction can be inferred from cytoplasmic SHH expression (arrows in **e’**) that con- verge on a focal point between mesenchymal tissue (asterisk in **e’**). IHH expression is very low (**b**) or absent (**d,f**) from distal phallic urethral epithelium, though expression is observed in rectal epithelium (**d’**, arrow). (**g**) Day 60 proximal phallus section as reference for images of sequential sections in h–i. (**h**) SHH expression predominates in cells in an intermediate position within the epithelium (asterisk), while (**i**) IHH expression is restricted to cells closer to the luminal edge and in cells with low SHH expression (arrows in 2**h’**–**i’**). (**j**–**k**) Day 90 proximal phallus sections with a similar pattern of expression as at day 60. (**l**–**m**) Longitudinal sections of day 90 phallus showing SHH expression throughout the epithelium but low in the outermost cell populations. (**k**) IHH expression observed in regions with low SHH (asterisks in **l**–**m**). (**n**–**o**) Distal phallus sections from Day 150 phallus treated with estradiol showing extensive SHH expression (**n**) but absence of expression in regions dominated by IHH expression (**o**). Small image inserts in e and f show sections labeled using isotype control antibodies. (ur = urethra; ps = phallic skin; scale = 100 μm).

As bone differentiation ontologies were most highly enriched when comparing male versus female phallus tissue, to determine whether SHH and IHH may have spatially-restricted functions in the developing phallus and urethra—as they do in bone development and repair—confocal immunofluorescence was performed to localise their expression through phallus development. Normal wallaby phallus tissue was immunolabelled for either SHH or IHH (Figure 2, red) in combination with the mesenchymal marker vimentin (Figure 2, green). In the distal phallus (Figure 2a–f), expression of SHH was observed throughout the urethral epithelium at all stages tested (Figure 2a–a’ (d20), 2c–c’ (d60), 2e–e’ (d90), asterisks). In contrast, IHH expression was either very low or undetectable in distal urethral plate epithelial cells (Figure 2b–b’ (d20), 2d–d’ (d60), 2f–f’ (d90)). However, IHH is known to be highly expressed in the gastrointestinal tract [26,27]. In Figure 2c–c’, the plane of sectioning captured the rectum. As an internal positive control, strong IHH expression is observed in the most apical cells of the rectal epithelium. Notably, the anogenital epithelium with the lowest SHH expression (Figure 2c’, arrow) coincides with that expressing high levels of IHH (Figure 2d’, arrow). In addition, under high z-axis resolution it was possible to observe an ordered arrangement of urethral plate epithelial cells, with cytoplasmic SHH directed towards regions at a critical juncture between mesenchymal cells and urethral plate epithelium (Figure 2e’, arrows pointing to a “focal point” marked by an asterisk).

In contrast to the distal phallic epithelium, expression of IHH was observed in a small proportion of proximal phallic urethral epithelial cells. To determine whether IHH and SHH were expressed in the same population of cells, sequential sections were assessed at high magnification and z-axis resolution (low magnification image in Figure 2g, dotted box denotes area analysed at high magnification in Figure 2h,i). This revealed that SHH and IHH were expressed in separate areas, with SHH expression highest in cells with an intermediate position within the epithelium (Figure 2h, asterisk), while IHH expression was concentrated to cells nearer to the lumen edge (Figure 2i’, arrows). This segregated expression pattern was most clearly observed in the perineum (Figure S1).

This pattern was also observed in the proximal phallus of d90 animals (Figure 2j,k). Longitudinal sections of the phallus at d90 confirmed that SHH protein was present throughout the urethral epithelium and urethral plate epithelium at this developmental stage, though with differing intensities in different regions (Figure 2l). In contrast, IHH expression was restricted to the outermost layers of cells in the urethral plate and phallic skin (Figure 2m). This pattern of protein localisation was also observed at d140 (Figure 2n,o, asterisks). Overall, SHH and IHH were expressed in different tissue regions within the wallaby phallus epithelium, where SHH was associated with intermediate regions within the epithelium and IHH expression restricted to more luminal cell layers.

### 2.3. Specific Hedgehog Proteins Elicited Discrete Gene Expression Changes in Developing Phallus

**Figure 3:**
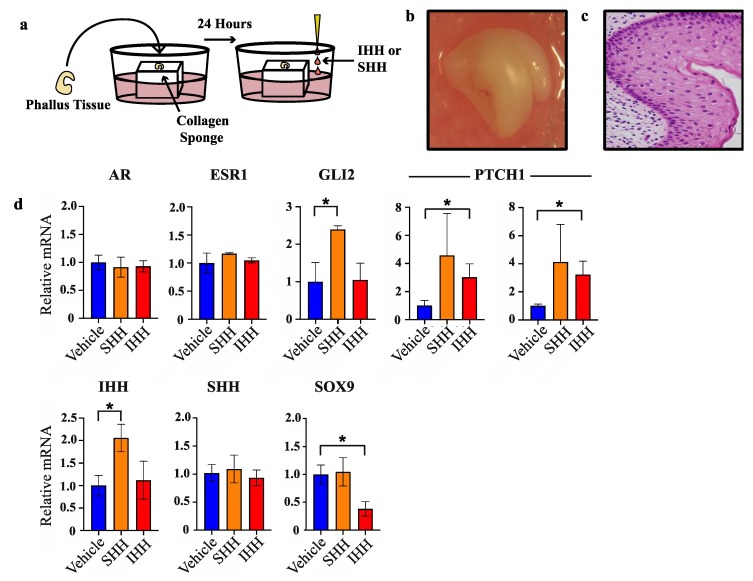
SHH and IHH elicit different transcriptional responses in phallus explant culture. (**a**) Explant culture protocol. (**b**) Gross appearance of phallus tissue after 72 h in culture. (**c**) Haematoxylin and esoin stain of PFA-fixed tissue section of phallus tissue in b. (**d**) Relative mRNA expression in phallus explants as determined by qPCR (*n* = 3, mean +/− s.d, * = <0.05).

To test whether the segregated expression pattern of SHH and IHH reflects separate molecular functions of these factors in the developing phallus, normal day 60 phallus tissue was cultured as explants and treated with either SHH or IHH for 24 h as outlined in Figure 3a. This developmental stage was selected as it is outside of the androgen programming window and at the very beginning of sexual dimorphic phallus development and urethral closure in the wallaby. After explant treatment, tissue was collected for RNA extraction and mRNA quantification by qPCR. Next it was investigated whether SHH and IHH can act to modulate expression of known regulators of urethral development, such as androgen and oestrogen receptor, as well as a known direct target of SHH in developing bone, the transcription factor SOX9 that itself plays important roles in sexual differentiation.

Whole organ or histological staining of phallus tissue cultured for 72 h showed no obvious tissue necrosis or cell death (Figure 3b and Figure 3c, respectively). Treatment with either SHH or IHH failed to induce changes in oestrogen receptor (*ESR1*) or androgen receptor (*AR)* mRNA when compared to vehicle treated tissue (Figure 3d). In contrast, while SHH treatment induced a significant induction of *GLI2* transcription—a result confirming its in vitro activity—no increase in *GLI2* transcription was observed after treatment with IHH. However, IHH treatment induced a clear increase in *PTCH1* transcription, another SHH/IHH target gene. SHH treatment caused increases in *PTCH1* transcription that were highly variable and did not reach statistical significance. Upon replication with an additional primer set similar results were observed that reflects a likely biological or sampling source for this variation.

Treatment with SHH induced a significant increase in *IHH* transcription (2.1-fold, *n* = 3, *p* < 0.04) but no change in *SHH* or *SOX9*. In contrast, IHH treatment induced a significant decrease in *SOX9* transcription (2.6-fold, *n* = 3, *p* < 0.02) but did not alter the levels of *IHH* or *SHH* transcription. These findings show that at this early stage of urethral closure in the tammar wallaby, SHH induced *IHH* transcription while IHH suppressed *SOX9* transcription.

### 2.4. Positive Association between SOX9 and SHH Protein Expression, and Negative Association between SOX9 and IHH Expression, in Normal Phallus Tissue

**Figure 4:**
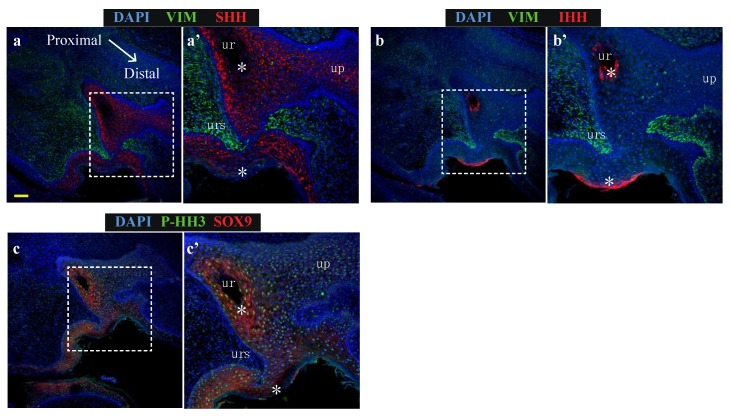
SHH and SOX9 expression is low in urethral epithelial and phallic skin cells expressing IHH (**a**) Expression of SHH (red) and vimentin (green), (**b**) Expression of IHH (red) and vimentin (green) or (**c**) Expression of SOX9 (red) and proliferation marker, P-HH3 (green) in oblique sections of day 60 wallaby phallus tissue. (ur = urethra; up = urethral plate epithelium) Scale = 100 μm.

Findings in Figure 3 indicate that SHH and IHH have different actions on transcription in the developing phallus. To determine whether these findings are relevant on a tissue and protein level, immunolocalisation for IHH, SHH or SOX9 was performed on sequential oblique sections from normal d60 phallus tissue (Figure 4), to capture both a closed portion of the urethra and a component of the urethral plate epithelium.

Consistent with Figure 2, SHH localised along the entire urethral epithelium and urethral plate epithelium but was absent from two distinct regions of epithelial cells: the luminal-most cells in the closed urethra as well as regions in the outermost cell layers of the external penile skin (Figure 4a’, asterisks). The expression pattern of epithelial IHH was broadly inverse to the expression pattern of SHH, with IHH expression identified only in the most luminal cells of the closed urethra and the outermost cell layer of phallus skin (Figure 4b’, asterisks). Consistent with findings from explant cultures of reduced SOX9 transcription after treatment with IHH, regions expressing high IHH exhibit very low SOX9 expression (Figure 4c’, asterisks). In these regions with low SOX9 there was significantly lower levels of the proliferation marker phosphorylated histone H3 (P-HH3: Figure 4c’, asterisks). Therefore, the ability of purified IHH to induce a reduction in *SOX9* transcription in phallus explant culture is supported by their protein localisation patterns at single cell level in vivo. Furthermore, expression of high epithelial IHH is associated with cells that do not express a marker of proliferation.

## 3. Discussion

This study is the first in any species to identify that specific hedgehog proteins have distinct patterns of localisation and effects on gene expression in the developing phallus. Such region-specific expression and action of SHH and IHH led us to formulate a new model of urethral closure whereby the expression of IHH antagonises the actions of SHH, partly by inhibiting SOX9 expression. In this model, the coordinated timing of SHH and IHH expression at critical regions in the phallus is an important element of appropriate urethral development and closure. This conclusion is supported by the lack of proliferation in cells of the developing phallus that express IHH, while widespread proliferation was observed in SHH-expressing epithelial cells.

Results herein support other studies identifying gene regulatory networks present in bone/limb formation as being important in phallus development in a variety of species [3,4,5,6]. Furthermore, a comparative evolutionary study determined that a key bone structure of the phallus in many species, the baculum or os penis, has evolved a minimum of nine times and lost a minimum of 10 times in evolutionary history [28]. While both humans and wallabies lack an os penis, the fact such a structure has re-emerged many times provides a strong indication that the signalling networks involved in chondrogenesis and bone formation persist in the developing phallus of the wallaby. It should not, therefore, be surprising that genes involved in early bone formation were identified as being differentially expressed between male and female, as well as male wallabies treated with E2. However, results of the present study take this perspective further by demonstrating that, far from being surrogates, SHH and IHH likely play critically distinct roles during urethral closure that may mimic signalling networks present in developing bone. This is supported by their expression in discrete tissue compartments, and their ability to elicit different transcriptional responses in a key regulator of cell/tissue development, SOX9. A critical role is played by SOX9 during gonad as well as cartilage and bone development. Removal of SOX9 from SHH-expressing cells failed to induce morphological changes in genital development [29], though SOX9 expression in the mouse GT shows a restricted pattern within a subset of urethral and preputial epithelium that may be akin to those observed in the study presented herein. Therefore, it is possible that the role of SOX9 is critical in the IHH-expressing population that will have escaped SHH-driven knockout of SOX9.

These findings break new ground in our mechanistic understanding of phallus development and urethral closure. In particular these findings indicate that contrary to current models that are limited to epithelial-mesenchymal tissue interactions in hedgehog signalling, the mechanisms underpinning urethral closure may also involves inter/intra-epithelial signalling. While a novel finding in the phallus, distinct expression patterns and functions of SHH and IHH are found in other tissues. For example during craniofacial suture morphogenesis [30,31], as well as during tendon development, attachment and migration [32], where SHH and IHH are believed to occupy distinct tissue regions and play distinct temporal functions during development.

The restricted localisation of IHH protein to the luminal most cells in the proximal penile urethral, perineum and rectum/gastrointestinal tract may reflect an endodermal origin of these cells, that is consistent with a model of urethral closure whereby the urorectal septum divides the urogenital and anorectal tracts [33]. In that model, endodermal and ectodermal tissue fuse along the length of the phallus during urethral closure with the ectodermal cells contributing to the outermost layers of a subset of developing urethral epithelium and penile skin. It is possible that the interactions of IHH and SHH during phallus development described through results of the study presented herein, serve as the signalling network regulating the interactions of these two tissues during urethral closure. Such a model adds an extra dimension to our understanding of the cell and tissue interactions regulating urethral closure.

Overall this study demonstrated evidence of a novel mechanism to explain the process of urethral closure during phallus development, providing important new direction for researchers aiming to understand the tissue signalling networks underpinning phallus development and urethral closure.

## 4. Materials and Methods

### 4.1. Animal Treatments and Tissue Collection

Tammar wallabies were collected from wild populations originating on Kangaroo Island (South Australia) and were held in a breeding colony in Melbourne (Victoria) for breeding and experimentation. Females were monitored from 25–30 days after removal of a pouch young that initiates reactivation of their diapause blastocysts, to check for newborns [34]. The sex of newborns was determined by the presence of scrotal or mammary primordia, as previously described. For young of unknown day of birth, this was estimated from measurements of head length and weight from established growth curves [35]. Pouch young were treated from days 20–40 after birth, and treatments were performed as previously described [36].

Phallus tissues were collected from tammar wallaby pouch young after anaesthesia with Zoletil^Ⓡ^ 100 (Lyppard Australia Cat# ZOLED (Tiletamine HCl 50 mg/mL, Zolazepam HCl 50 mg/mL, used at 1 mL/kg)). Samples were snap-frozen and stored at −80 °C for RNA extraction or immersion-fixed in 4% paraformaldehyde for histological analysis. All animals were treated and tissues collected under appropriate permits, and experiments approved by the University of Melbourne Animal Experimentation Ethics Committees in accordance with the National Health and Medical Research Council of Australia animal ethics guidelines.

### 4.2. Explant Culture and Treatment

Collagen dental sponges (10 cm cubes—(Dental Solutions Israel, Israel, Cat# DSP-32)) were immersed in DMEM plus 10% fetal calf serum (Thermo-Fisher, Waltham MA, USA, Cat#s 11995115 and 10100147, respectively) until saturated. Dissected phallus tissue from d60 male pouch young were cultured atop saturated sponges for 24 h at 37 °C for tissue acclimatisation. After this, media was removed and replaced with DMEM plus 10% fetal calf serum, with the addition of either vehicle (ethanol—0.25%), SHH (0.25 ug/mL—Abcam, Cambridge, UK, #Ab123773) or IHH (0.25 ug/mL—Abcam, Cambridge, UK, #Ab243268) and media allowed to equilibrate for 5 min at 37 °C. This was subsequently replaced with fresh media supplemented with SHH or IHH and culture for an additional 24 h. Phallus tissue was subsequently snap-frozen for RNA extraction.

### 4.3. RNA Extraction and Next Generation Sequencing

RNA material was extracted and prepared for sequencing as previously described [37]. Briefly, RNA was subjected to multiplex indexed-RNAseq analyses using TruSeq kit (Illumina, San Diego, CA, USA) and a HiSeq2500 analyzer (Illumina). Roughly 10–14 × 10^6^ reads (single end 100 bp) were obtained from each sample after Q.V.>30 filtering. RNA sequencing data were analysed with FastQC followed by CutAdapt to remove bases sequenced with high uncertainty. Mapping was performed With RNA Star [38] and the annotated tammar wallaby genome 3.0 (Heider et al., personal communications). The number of reads for each annotated gene was determined using FeatureCounts. Differential gene expression analysis was executed using DESeq2 [39].

### 4.4. RNA Extraction for cDNA Generation and qPCR Analysis

Frozen phallus tissues were digested in TriZOL^Ⓡ^ reagent (Thermo Fisher Scientific, Waltham MA, USA, Cat# 15596026) using an IKA T10^Ⓡ^ basic homogeniser (Lab Gear, Australia, Cat# IKAW3737000), followed by chloroform extraction as per manufacturer’s instructions using a PureLink RNA mini kit (Thermo Fisher Scientific, Waltham MA, USA, Cat# 12183018A). Contaminating DNA was removed using DNase (Ambion, Austin, TX, USA Cat# 1906) and the manufacturer’s protocol. For cDNA synthesis, 500ng of RNA was used as template employing the SuperScript IV^Ⓡ^ cDNA synthesis kit (Thermo Fisher Scientific, Waltham MA, USA, Cat# 18091050). For quantification of cDNA, SsoAdvanced^Ⓡ^ Universal SYBR Green Supermix was employed (BioRad, Hercules, CA, USA, Cat# 1725272) utilising primers for AR, ESR1, SHH, IHH, SOX9 and the housekeeping genes GAPDH and 18S (see Table 1). A 384-well QuantStudio^Ⓡ^ 5 thermal light cycler (Thermo Fisher Scientific, Waltham MA, USA) was employed for quantification of SYBR intensities. The standard curve method was employed for relative quantification of cDNA.

### 4.5. Immunofluorescence Microscopy

Wallaby young were removed from the pouch and phallus tissue dissected and placed in 4% paraformaldehyde overnight at 4 °C on a carousel. Tissue was washed for 2 × 30 min in PBS (pH7.4) at room temperature followed by transfer to 70% ethanol for tissue processing. Tissue was processed and embedded in paraffin wax, sectioned at 5um thickness before mounting on Superfrost^Ⓡ^ ultra PLUS slides (Thermo-Fisher Scientific, Waltham MA, USA). Tissue dewaxing, rehydration, antigen retrieval, primary and secondary antibody staining protocols were performed as previously described [40], with an additional blocking step being added between antigen retrieval and primary antibody incubation (see Table 2 for antibody incubation conditions). This blocking step involved incubation of slides with 0.3% Sudan black (ProSciTech, Qld, Australia) in 70% ethanol for 10 min. This was followed by brief immersion in fresh 70% ethanol before incubation for 5 min in PBS and primary antibody incubation. Images were acquired on a Nikon (Minato City, Japan) A1R^Ⓡ^ spectral confocal microscope. Specificity of the primary antibody was determined by staining positive and negative control tissue, as well as substitution of the target-specific primary antibodies with non-specific IgG control antibodies. All 40x and 63x images were acquired at minimum airy unit values to maximise z-axis resolution.

### 4.6. Haematoxylin and Eosin Staining of Explant-Cultured Phallus Tissue

Explant tissue was processed into paraffin blocks, sectioned, dewaxed and rehydrated as per immunofluorescence protocol. Sections were then immersed in Mayer’s haematoxylin (Sigma-Aldrich, St Louis MS, USA) for 5 min, washed in tap water for 1 min, followed by incubation in 0.3% acid ethanol for 1 min, washing in tap water for 1 min, immersion in 1% eosin (Sigma,-Aldrich, St Louis MS, USA) in 75% ethanol and a final 1 min wash in running tap water. This was followed by dehydration in graded ethanol, clearing in histolene and mounting with UltraMount No. 4 (Thermo-Fisher Scientific, Waltham MA, USA).

## Figures and Tables

**Table 1:** Sequences of qPCR primers used in Figure 4.

Target Gene	Sequences
ACTB	F – TTGCTGACAGGATGCAGAAG R – AAAGCCATGCCAATCTCATC
AR	F – CACATTGAAGGCTATGCGTG R – CCCATCCAGGAGTACTGAAT
ESR1	F – TGATCAACTGGGCAAAGAGGG R – GATGTAGCCAGCAACATGTCA
GAPDH	F – TCCCAATGTATCTGTTGTGGATCTG R – AACCATACTCATTGTCATACCAAGAAAT
GLI2	F – GTTCACAGCTAGTGGCTCC R – ACTGCTGCCTCACTGCTTTG
IHH	F – CTTCCTGGCCTTCTTGGACC R – CTTCCTGGCCTTCTTGGACC
PTCH1 #1	F – AATGAAGACAAGGCAGCAG R – TAGCAACTCGGATAACACT
PTCH1 #2	F – AATGACTCCCAAGCAAATGTA R – TAGACAGGCATAGGCGAGCAT
SHH	F – CTTCCTGGCCTTCTTGGACC R – CTTCCTGGCCTTCTTGGACC
SOX9	F – TGCGAGTCAATGGCTCTAGCAA R – CTCCTCCGAGGTTGGTATTTGT

**Table 2:** Primary antibodies and incubation conditions.

Target Protein	Supplier and Catalogue Number	Dilution
IHH	LS Bio, Seattle, WA, USA, # LS-40514	1:400
SHH	Abcam, Cambridge, UK, # Ab19897	1:200
SOX9	Merck, Kenilworth, NJ, USA # Ab5535	1:800
Vimentin	Abcam, Cambridge, UK, # Ab8069	1:400

## Supplementary Materials

Supplementary materials can be found at https://www.mdpi.com/1422-0067/21/4/1237/s1.

## Abbreviations

ACAN	Aggrecan
AR	Androgen Receptor
BMP5	Bone morphogenic protein 5
E2 ESR1 FMOD IHH SHH	Oestradiol Oestrogen Receptor Alpha Fibromodulin Indian Hedgehog Sonic Hedgehog

## References

[1] Nassar N., Bower C., Barker A. (2007). Increasing prevalence of hypospadias in Western Australia, 1980–2000. Arch. Dis. Child..

[2] Springer A., Heijkant M.V.D., Baumann S., Information P.E.K.F.C. (2016). Worldwide prevalence of hypospadias. J. Pediatr. Urol..

[3] O’Shaughnessy K.L., Dahn R.D., Cohn M.J. (2015). Molecular development of chondrichthyan claspers and the evolution of copulatory organs. Nat. Commun..

[4] Tschopp P., Sherratt E., Sanger T.J., Groner A.C., Aspiras A.C., Hu J.K., Pourquie O., Gros J., Tabin C.J. (2014). A relative shift in cloacal location repositions external genitalia in amniote evolution. Nature.

[5] Herrera A.M., Cohn M.J. (2014). Embryonic origin and compartmental organization of the external genitalia. Sci. Rep..

[6] Infante C.R., Mihala A.G., Park S., Wang J.S., Johnson K.K., Lauderdale J.D., Menke D.B. (2015). Shared Enhancer Activity in the Limbs and Phallus and Functional Divergence of a Limb-Genital cis-Regulatory Element in Snakes. Dev. Cell.

[7] Perriton C.L., Powles N., Chiang C., Maconochie M.K., Cohn M.J. (2002). Sonic hedgehog Signaling from the Urethral Epithelium Controls External Genital Development. Dev. Boil..

[8] Seifert A.W., Bouldin C.M., Choi K.-S., Harfe B.D., Cohn M.J. (2009). Multiphasic and tissue-specific roles of sonic hedgehog in cloacal septation and external genitalia development. Development.

[9] Seifert A.W., Zheng Z., Ormerod B.K., Cohn M.J. (2010). Sonic hedgehog controls growth of external genitalia by regulating cell cycle kinetics. Nat. Commun..

[10] Haraguchi R., Suzuki K., Murakami R., Sakai M., Kamikawa M., Kengaku M., Sekine K., Kawano H., Kato S., Ueno N. (2000). Molecular analysis of external genitalia formation: The role of fibroblast growth factor (Fgf) genes during genital tubercle formation. Development.

[11] Miyagawa S., Matsumaru D., Murashima A., Omori A., Satoh Y., Haraguchi R., Motoyama J., Iguchi T., Nakagata N., Hui C.-C. (2011). The role of sonic hedgehog-Gli2 pathway in the masculinization of external genitalia. Endocrinology.

[12] Carmichael S.L., Ma C., Choudhry S., Lammer E.J., Witte J.S., Shaw G.M. (2013). Hypospadias and genes related to genital tubercle and early urethral development. J. Urol..

[13] Zheng Z., Armfield B.A., Cohn M.J. (2015). Timing of androgen receptor disruption and estrogen exposure underlies a spectrum of congenital penile anomalies. Proc. Natl. Acad. Sci. USA.

[14] Bouty A., Ayers K.L., Pask A., Héloury Y., Sinclair A.H. (2015). The Genetic and Environmental Factors Underlying Hypospadias. Sex. Dev..

[15] Dean A., Smith L.B., MacPherson S., Sharpe R.M. (2012). The effect of dihydrotestosterone exposure during or prior to the masculinization programming window on reproductive development in male and female rats. Int. J. Androl..

[16] Ittiwut C., Pratuangdejkul J., Supornsilchai V., Muensri S., Hiranras Y., Sahakitrungruang T., Watcharasindhu S., Suphapeetiporn K., Shotelersuk V. (2017). Novel mutations of the SRD5A2 and AR genes in Thai patients with 46, XY disorders of sex development. J. Pediatr. Endocrinol. Metab..

[17] Matsushita S., Suzuki K., Murashima A., Kajioka D., Acebedo A.R., Miyagawa S., Haraguchi R., Ogino Y., Yamada G. (2018). Regulation of masculinization: Androgen signalling for external genitalia development. Nat. Rev. Urol..

[18] Murakami R. (1987). A histological study of the development of the penis of wild-type and androgen-insensitive mice. J. Anat..

[19] Welsh M., MacLeod D.J., Walker M., Smith L.B., Sharpe R.M. (2010). Critical androgen-sensitive periods of rat penis and clitoris development. Int. J. Androl..

[20] MacLeod D.J., Sharpe R.M., Welsh M., Fisken M., Scott H.M., Hutchison G.R., Drake A.J., Driesche S.V.D. (2010). Androgen action in the masculinization programming window and development of male reproductive organs. Int. J. Androl..

[21] Sinclair A.W., Cao M., Pask A., Baskin L., Cunha G.R. (2017). Flutamide-induced hypospadias in rats: A critical assessment. Differ..

[22] Driesche S.V.D., Kilcoyne K.R., Wagner I., Rebourcet D., Boyle A., Mitchell R., McKinnell C., MacPherson S., Donat R., Shukla C.J. (2017). Experimentally induced testicular dysgenesis syndrome originates in the masculinization programming window. JCI Insight.

[23] Shao M., Ghosh A., Cooke V.G., Naik U.P., Martin-DeLeon P.A. (2008). JAM-A is present in mammalian spermatozoa where it is essential for normal motility. Dev. Biol..

[24] Sinclair A.W., Cao M., Baskin L., Cunha G.R. (2016). Diethylstilbestrol-induced mouse hypospadias: “window of susceptibility”. Differentation.

[25] Eden E., Navon R., Steinfeld I., Lipson D., Yakhini Z. (2009). GOrilla: A tool for discovery and visualization of enriched GO terms in ranked gene lists. BMC Bioinform..

[26] Brink G.R.V.D. (2007). Hedgehog Signaling in Development and Homeostasis of the Gastrointestinal Tract. Physiol. Rev..

[27] Brink G.R.V.D., Bleuming S.A., Hardwick J.C.H., Schepman B.L., Offerhaus G.J., Keller J.J., Nielsen C., Gaffield W., Van Deventer S.J.H., Roberts U.J. (2004). Indian Hedgehog is an antagonist of Wnt signaling in colonic epithelial cell differentiation. Nat. Genet..

[28] Schultz N.G., Lough-Stevens M., Abreu E., Orr T., Dean M.D. (2016). The Baculum was Gained and Lost Multiple Times during Mammalian Evolution. Integr. Comp. Boil..

[29] Sreenivasan R., Gordon C.T., Benko S., De Iongh R., Bagheri-Fam S., Lyonnet S., Harley V. (2017). Altered SOX9 genital tubercle enhancer region in hypospadias. J. Steroid Biochem. Mol. Boil..

[30] Lenton K., James A.W., Manu A., Brugmann S.A., Birker D., Nelson E.R., Leucht P., Helms J.A., Longaker M.T. (2011). Indian hedgehog positively regulates calvarial ossification and modulates bone morphogenetic protein signaling. Genesis.

[31] Santagati F., Rijli F.M. (2003). Cranial neural crest and the building of the vertebrate head. Nat. Rev. Neurosci..

[32] Felsenthal N., Rubin S., Stern T., Krief S., Pal D., Pryce B.A., Schweitzer R., Zelzer E. (2018). Development of migrating tendon-bone attachments involves replacement of progenitor populations. Development.

[33] Seifert A.W., Harfe B.D., Cohn M.J. (2008). Cell lineage analysis demonstrates an endodermal origin of the distal urethra and perineum. Dev. Boil..

[34] Wai-Sum O., Short R.V., Renfree M.B., Shaw G. (1988). Primary genetic control of somatic sexual differentiation in a mammal. Nature.

[35] Poole W.E., CSIRO (1991). Division of Wildlife and Ecology, Tables for Age Determination of the Kangaroo Island Wallaby (Tammar), Macropus Eugenii, from Body Measurements.

[36] Coveney D., Shaw G., Renfree M.B. (2001). Estrogen-induced gonadal sex reversal in the tammar wallaby. Boil. Reprod..

[37] Chen Y., Yu H., Pask A.J., Fujiyama A., Suzuki Y., Sugano S., Shaw G., Renfree M.B. (2018). Hormone-responsive genes in the SHH and WNT/beta-catenin signaling pathways influence urethral closure and phallus growth. Biol. Reprod..

[38] Dobin A., Davis C.A., Schlesinger F., Drenkow J., Zaleski C., Jha S., Batut P., Chaisson M., Gingeras T.R. (2013). STAR: Ultrafast universal RNA-seq aligner. Bioinformatics.

[39] Love M.I., Huber W., Anders S. (2014). Moderated estimation of fold change and dispersion for RNA-seq data with DESeq2. Genome Biol..

[40] Tarulli G.A., Stanton P.G., Lerchl A., Meachem S.J. (2006). Adult Sertoli Cells Are Not Terminally Differentiated in the Djungarian Hamster: Effect of FSH on Proliferation and Junction Protein Organization1. Boil. Reprod..

