# Supplementary material for: Discrete Hedgehog Factor Expression and Action in the Developing Phallus
Source: Int J Mol Sci. 2020 Feb 12;21(4):1237. doi: 10.3390/ijms21041237 (PMC7072906; doi:10.3390/ijms21041237)
Supplement: Supplementary file 1 [file ijms-21-01237-s001.pdf]

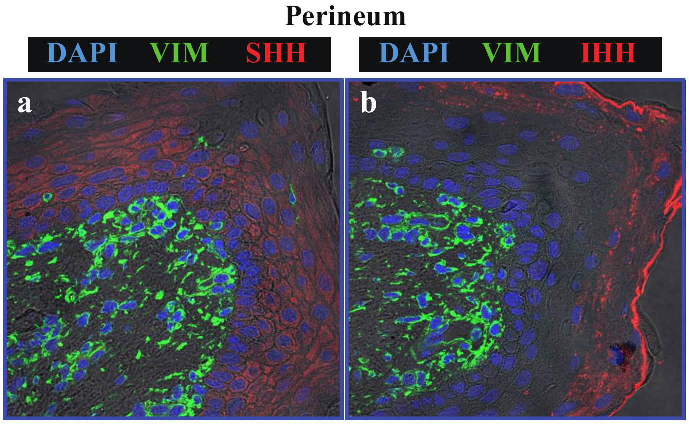

Supplementary Figure 1. Segregation of SHH and IHH expression in perineum skin of d60 wallaby. (**a**) Expression of SHH (red) and vimentin (green), or (**b**) Expression of IHH (red) and vimentin (green) in sagittal sections of day 60 wallaby phallus tissue.
